# Neurobiological After-Effects of Low Intensity Transcranial Electric Stimulation of the Human Nervous System: From Basic Mechanisms to Metaplasticity

**DOI:** 10.3389/fneur.2021.587771

**Published:** 2021-02-15

**Authors:** Sohaib Ali Korai, Federico Ranieri, Vincenzo Di Lazzaro, Michele Papa, Giovanni Cirillo

**Affiliations:** ^1^Division of Human Anatomy – Laboratory of Neuronal Networks, University of Campania “Luigi Vanvitelli”, Naples, Italy; ^2^Neurology Unit, Department of Neurosciences, Biomedicine and Movement Sciences, University of Verona, Verona, Italy; ^3^Neurology, Neurophysiology and Neurobiology Unit, University Campus Bio-Medico, Rome, Italy; ^4^ISBE Italy, SYSBIO Centre of Systems Biology, Milan, Italy

**Keywords:** transcranial direct current stimulation, transcranial alternating current stimulation, neurobiological after-effects, synaptic plasiticty, non-invasive brain stimulation

## Abstract

Non-invasive low-intensity transcranial electrical stimulation (tES) of the brain is an evolving field that has brought remarkable attention in the past few decades for its ability to directly modulate specific brain functions. Neurobiological after-effects of tES seems to be related to changes in neuronal and synaptic excitability and plasticity, however mechanisms are still far from being elucidated. We aim to review recent results from *in vitro* and *in vivo* studies that highlight molecular and cellular mechanisms of transcranial direct (tDCS) and alternating (tACS) current stimulation. Changes in membrane potential and neural synchronization explain the ongoing and short-lasting effects of tES, while changes induced in existing proteins and new protein synthesis is required for long-lasting plastic changes (LTP/LTD). Glial cells, for decades supporting elements, are now considered constitutive part of the synapse and might contribute to the mechanisms of synaptic plasticity. This review brings into focus the neurobiological mechanisms and after-effects of tDCS and tACS from *in vitro* and *in vivo* studies, in both animals and humans, highlighting possible pathways for the development of targeted therapeutic applications.

## Introduction

In the last two decades, therapeutic efficacy of non-invasive transcranial brain stimulation techniques through low-intensity electrical fields has been demonstrated by a number of works and clinical trials providing promising results for many neurological disorders, including stroke (1) and epilepsy (2, 3), movement disorders/Parkinson's (PD) (4) and Alzheimer's (AD) (5, 6). Due to non-invasiveness and transient side effects (7), transcranial electrical stimulation (tES) has found progressively a wide field of applications. Moreover, acquisition of recent experimental data has extended our knowledge of the cellular and molecular mechanisms involved in the after-effects of tES, thus supporting its therapeutic potential for brain disorders based on impaired synaptic plasticity (2).

The basic principle of tES is very simple and based on the negative (anodal) and positive (cathodal) currents and their flow into the brain (8). However, neurobiological mechanisms and after-effects are not yet fully understood. Experimental evidence has demonstrated that weak low-intensity ES (at an intensity lower than that needed for triggering action potentials) induces polarity-specific changes in spontaneous and evoked neuronal activity (9, 10): anodal polarization increases neuronal activity, whereas cathodal polarization decreases it (11–14). Accordingly, transcranial direct current stimulation (tDCS) has been shown to induce long-lasting and polarity-specific changes of human motor cortex excitability (15–17) related to modifications of synaptic efficacy similar to those underlying long-term potentiation (LTP) and long-term depression (LTD) of synaptic activity (18, 19). Studies of the effects of direct current stimulation (DCS) in slices of mouse primary motor cortex have shown that anodal DCS, in the absence of simultaneous synaptic activation, does not induce LTP/LTD like changes but it can modulate LTP induction (20). In contrast, by coupling DCS with low frequency stimulation (at 0.1 Hz), a long-lasting polarity- (anodal DCS) and frequency- specific LTP is obtained, mainly depending on N-methyl-D- aspartate (NMDA) receptor activation and secretion of brain-derived neurotrophic factor (BDNF) (21). In summary, these studies highlight the complex nature of tDCS effects, characterized by the capability of inducing and modulating LTP/LTD. However, while the immediate effects of tES can be explained by changes in transmembrane potential influencing neuronal firing, it is plausible that the long-term after-effects are likely due to modifications of intracellular calcium dynamics and mechanisms of synaptic plasticity, based on LTP/LTD processes (18, 22, 23) and/or induction of metaplasticity, the activity-dependent physiological changes that modulate neural plasticity (24). Anodal tDCS, for example, induces neurotrophic BDNF-mediated priming after-effects on synaptic plasticity and memory, making synapses susceptible to LTP induction in the rat hippocampus (25).

This work aims to comprehensively summarize the neurobiological mechanisms of tES and discuss future clinical applications. In particular, we first analyzed the technical aspects of electrical stimulation techniques, and then the neurobiological after-effects of tES on the constituents of the synaptic structure, distinguishing those on membrane polarity, neural transmission, synaptic plasticity, neuronal network and connectivity, and finally the effects on glial cells and neuroinflammation.

We believe that understanding the basis of the modulatory effect of tES would be particularly relevant for its clinical application in humans, where it could be used to shape the plastic properties of the brain.

## Technical Aspects: Transcranial Current and Magnetic Stimulation

According to whether direct or alternating current is applied to the brain, the method is referred to as either transcranial direct current stimulation (tDCS) or transcranial alternating current stimulation (tACS). Both techniques produce effects on cortical excitability outlasting the stimulation, up to 3 h with tDCS (26) and up to 1 h with high-frequency tACS (27–29). TDCS acts in a polarity-dependent fashion, with anodal stimulation increasing and cathodal stimulation decreasing neuronal excitability, whereas tACS consists in the application of a sinusoidal waveform current that alternates between the anode and the cathode (*switching polarity*) and modulates the power of oscillatory rhythms in a frequency-dependent manner by synchronizing or desynchronizing neuronal networks (30). For example, in studies that coupled transcranial magnetic stimulation (TMS) with ES, tACS was found to synchronize cortical networks bursting at frequencies higher than 300 Hz (31).

The association between the type of stimulation and neural response depends on many physical properties including the electrode type, length, strength, and frequency of stimulation (32). Low-intensity, constant, or non-constant currents are used for tDCS and delivered in rectangular or sinusoidal waves with pulses of unidirectional current, whilst non-constant current is used for tACS (33). TDCS flows into the brain from a battery-powered generator through a couple of sponge electrodes, with one or both the electrodes fixed over the scalp. It has been demonstrated that current density (i.e., current intensity/electrode size), duration, polarity, and location of stimulating electrodes have important implications in the modulatory outcome of stimulation (34). Generally, tDCS does not involve synaptic effects but polarity changes of the membrane resting potential, does not induce neuronal firing but rather modulates spontaneous neuronal network activity, polarizing brain tissue (35–37). The two types of stimulation, anodal and cathodal, do not contrast each other in terms of after-effects and modulation of their intensity dramatically produces different results. Generally, the cortical excitability is increased by anodal tDCS while it is decreased by the cathodal tDCS over the same area (site specificity).

TACS is a non-constant current which alternates its pulses with the opposite amplitude (38, 39). Despite site specificity, its effects are not site limited as tACS influences other areas of the brain through interneuronal circuits (33) and directly interferes with ongoing brain oscillations (40). TACS shares the same setup of tDCS: it is applied between electrodes placed over the target scalp sites, with intensity in the same range of 1–2 mA. The physiological bases of tACS are less explored than tDCS. The main biophysical (electric field strength and spatial distribution) and polarizing properties of tDCS should also apply to tACS, with the main difference that the polarity (i.e., the direction of current flow) changes of 180° during each cycle of the sinusoidal waveform of tACS and that the maximum current flow is present only at the peak of the alternating current.

The advantage of tACS is that it allows the manipulation of amplitude, frequency, and coherence of intrinsic neuronal oscillations (41, 42). In addition, the effects of tACS could be translated into whole larger brain-network activity through five different neuronal mechanisms (43, 44): (1) *stochastic resonance*, consisting in the stochastic response of tACS-affected neurons to be either polarized or hyperpolarized; (2) *rhythm resonance*, synchronizing tACS frequency with the endogenous oscillations; (3) *temporal biasing of spikes*, a synergistically excitation of the same groups of neurons during each cycle of stimulation; (4) *network entrainment* of an endogenous irregular neuronal activity that necessitates an external current with sufficiently stronger amplitude; (5) *imposed pattern*, tACS overcomes endogenous regular oscillations and introduce a new oscillation. These mechanisms attribute the large-scale effects of tACS to two synergistic phenomena: entrainment and neuroplasticity, respectively. The first takes place when an external rhythmic system affects another one, forcing it to follow its own oscillating frequency and phase; the second, through LTP/LTD phenomena, elicits offline tACS effects by increasing or decreasing neural synchronization, as confirmed by many studies (29, 45–47).

TACS has diverse modes of administration in terms of frequency: the beta (20 Hz), alpha (10–12 Hz), and gamma range (40 Hz), each producing diverse neurobiological effects for modulation of different bands of neural oscillations (42). The effects of alpha and gamma stimulation have been studied on attention with gamma stimulation demonstrating to facilitate endogenous attention (48).

Experimental and clinical applications of transcranial magnetic stimulation (TMS) is widely and progressively increased over the past two decades. In particular, several repetitive TMS (rTMS) protocols have been proved to modulate brain functions (from the molecular to the network scale) and human behavior (49, 50). For example, application of simple rTMS to a target cortical area for several minutes induces after-effects in a frequency- dependent manner (low frequency, ≤1 Hz, reduces cortical excitability whereas high-frequency, >5 Hz, does the opposite) (51) while theta-burst stimulation (TBS), a patterned protocol, induces longer-lasting effects with shorter application time (continuous TBS has primarily an inhibitory effect on corticospinal excitability, while intermittent TBS has an excitatory effect) (52).

TMS shares fundamental similarities with tES as both share neurobiological modulations at similar levels and involve rapid changes in magnetic fields (53). While TMS requires passing of current through coils to generate a magnetic field that in turn generates an electric field and a current density, in tES the electric field and the current density are proportional to injected current (54).

## Neurobiological After-Effects of Current Stimulation of Central Nervous System

### Effects on Membrane Polarity

Table 1 summarizes the results of the studies that analyzed the effects of tES on membrane polarity. Evidence has demonstrated that tDCS can modify neuronal membrane polarity and therefore the action potential generation (15, 19, 55) through activation of voltage-gated pre and postsynaptic Na^+^ and Ca^2+^ channels thus causing increased presynaptic release of excitatory neurotransmitters and postsynaptic calcium influx, respectively (15). Moderate but prolonged intracellular Ca^2+^ increase causes LTD while short but large Ca^2+^ increase causes LTP (64).

**Table 1:** tES after-effects on membrane polarity.

**References/Study**	**Methodology tES**	**Targets**	**Main results**
Nitsche and Paulus (15); Liebetanz (19); Stagg and Nitsche (55)	tDCS	Pre/post synaptic Na^+^ and Ca^2+^ channels	tDCS generates action potential via Na^+^ and Ca^2+^ channels by increasing presynaptic release of excitatory transmitters and Ca^2+^ influx
Zaghi et al. (33); Bikson et al. (56)	tDCS	Hippocampal neurons	Somatic polarization was obtained with electric field parallel to somato-dendritic axis in hippocampal neurons
Bikson et al. (56); Arlotti et al. (57); Rahman et al. (58); Pelletier and Cicchetti (32); Seo and Jun (59)	tDCS - aDCS - cDCS	Structural components of neurons	Components at the cathode depolarize while those at the anode hyperpolarize
Francis et al. (60); Deans et al. (61); Reato et al. (62)	tACS	Neuronal resonance	tACS can induce cumulative effects over multiple cycles that can shift in spike timing.
Bindman et al. (11); Bikson et al. (56); Antal and Herrmann (63)	tDCS - aDCS - cDCS	Transmembrane potentials	Constant electric field shifts neuronal transmembrane potential to less negative in cDCS and more negative in aDCS which makes it more prone to generate action potential.

***tES**, transcranial electrical stimulation; **tDCS**, transcranial direct current stimulation; **tACS**, transcranial alternating current stimulation; **a/c tDCS**, anodal/cathodal transcranial direct current stimulation*.

The polarity-dependent effect of tDCS is strictly dependent on the orientation of axons and dendrites (33). Specifically, when the effect of polarity was studied *in vitro* on hippocampal neurons (56), somatic polarization was obtained with the electric field parallel to the somato-dendritic axis, while an effect on afferents without somatic polarization was produced by the electric field perpendicular to the apical-dendritic axis. Moreover, the structural components of the cell at the cathode depolarize while the elements facing the anode are subject to hyperpolarization (32, 56–59). On the other hand, tACS, matching resonant neuronal properties, can induce cumulative effects over multiple cycles that may cause shift in spike timing (60–62).

However, these biophysical properties might produce complex modulatory effects when tES is applied to circuits of the human brain with no uniform spatial orientations. Based on experimental studies (11, 56), the applied constant electric field shifts the transmembrane potential of neurons toward less negative (anodal stimulation) or more negative values (cathodal stimulation), thus increasing or decreasing the likelihood of generation of action potentials (63), thus influencing both spontaneous and evoked neuronal firing.

### Effects on Neural Transmissions

Many studies have shown that tACS interferes with several neurotransmitter systems. The balance between cholinergic and adrenergic system after administration of reserpine (an anti-adrenergic drug that irreversibly blocks the H^+^-coupled vesicular monoamine transporters—VMAT) and physostigmine (a parasympathomimetic reversible cholinesterase inhibitor) occurred much faster while applying tACS: it was observed that the quantity of presynaptic vesicles first declined, then increased after 5 min and then returned to baseline levels after tACS (65). Evidence suggested that this type of stimulation might modulate the serotoninergic raphe nuclei, the noradrenergic locus coeruleus, the cholinergic latero-dorsal tegmental, and pedunculopontine nuclei in the brainstem (66). Additionally, tACS was found to modulate the levels of endorphins into the cerebrospinal fluid (67) and naloxone, a pure opioid antagonist, was reported to reduce tACS analgesic effects (67), prompting to hypothesize a tACS-induced modulation of the neurotransmitters' release.

The blockage of serotonin reuptake increases LTP in the motor cortex by anodal tDCS and shifts LTD to LTP after cathodal tDCS (68). In addition, anodal tDCS was demonstrated to reduce γ-aminobutyric acid (GABA) concentration in the stimulated cerebral cortex while cathodal tDCS impaired glutamatergic neuronal activity and reduced GABA concentration (2, 69). Authors argue that these protocols might be used therapeutically to reduce the imbalance between excitatory and inhibitory transmitters (70, 71). These results were also confirmed in humans by magnetic resonance spectroscopy (MRS) studies examining the effects of tDCS on the hand area of the primary motor cortex. Accordingly, authors reported that anodal tDCS causes GABA decrease while cathodal tDCS decreases both the levels of glutamate and GABA (70). Upon administration of GABA antagonists, anodal tDCS produces delayed but enhanced excitability increase in cortical or subcortical areas (72). See Table 2 for a summary of the studies that analyzed the effects of tES on neural transmissions.

**Table 2:** tES after-effects on neural transmission.

**References/Study**	**Methodology tES**	**Targets**	**Main results**
Kirsch and Nichols (65)	tACS	Cholinergic and adrenergic neural transmission	After administration of reserpine and physostigmine and administration of tACS, the quantity of presynaptic vesicles declines and then increased
Nitsche et al. (68)	tDCS - aDCS - cDCS	Motor cortex	Blockage of serotonin reuptake increases LTP via aDCS and shifts LTD to LTP after cDCS
Stagg et al. (70); Nitsche et al. (72)	tDCS - aDCS - cDCS	GABA and glutamate in cortical and subcortical areas	aDCS reduces GABA while cDCS reduces both glutamate and GABA. With GABA antagonists, aDCS produced enhanced excitability in cortical and subcortical areas

***tES**, transcranial electrical stimulation; **tDCS**, transcranial direct current stimulation; **tACS**, transcranial alternating current stimulation; **a/c tDCS**, anodal/cathodal transcranial direct current stimulation; **LTP**, long-term potentiation; **LTD**, long-term depression; **GABA**, gamma amino butirric acid*.

### Effects on Synaptic Plasticity

Experimental and human studies suggest that the after-effects of tES might originate from persistent modifications of synaptic efficacy similar to those underlying LTP and LTD of synaptic activity (18, 19, 73). Synaptic plasticity usually involves short- and long-term modifications of existing synapses (formation, removal, and remodeling of synapses and dendritic spines) that in turn modify the activity of brain networks in which they are interposed (50). Mechanisms of synaptic plasticity occur at different levels, from ultrastructural to synapse: calcium dynamics, neurotransmitter release, proteins (receptors, transporters, and ion channels) and gene expression (74). Table 3 summarizes the main results of the studies that analyzed the tES after-effects on synaptic plasticity.

**Table 3:** tES after-effects on synaptic plasticity.

**References/Study**	**Methodology tES**	**Targets**	**Main results**
Ranieri et al. (20)	tDCS - cDCS - aDCS	Neuronal LTP	aDCS increased LTP while cDCS decreased LTP
Fritsch et al. (21); Yu et al. (25)	tDCS	BDNF/TrkB signaling	tDCS increases BDNF concentration which induces LTP. TrkB stimulation by BDNF promotes late phase LTP
Lanté et al. (75); Luscher and Malenka (76)	tDCS	NMDA/AMPA receptors	High frequency stimulation induced LTP in active NMDA receptors, expression of AMPA receptors in postsynaptic neuron and Ca^2+^ rise. Low frequency stimulation induces small rise in Ca^2+^ and presynaptic internalization of AMPA by phosphatase activation and LTD generation
Mycielska and Djamgoz (77); McCaig et al. (78)	tDCS	Cellular migration	tDCS modified the speed and direction of cell migration by shifting intracellular Ca^2+^ and modifying expression of EGFR due to electrostatic effects
Monte-Silva et al. (79); Kuo et al. (80)	tDCS - cDCS - aDCS	L-DOPA induced plastic changes	Anodal L-DOPA suppressed plasticity induced by atDCS while prolonged the reduction of excitability by cDCS
Hurley and Machado (6)	tDCS	Neuronal polarity	When synaptic activity is preconditioned by tDCS, continuous tDCS after interval will modulate polarity
Carvalho et al. (81)	tDCS - aDCS - cDCS	Working memory	Continuous aDCS facilitates performance and cDCS enhances working memory
Zaehle et al. (45)	tACS	Rhythmic patterns and natural pattern	tACS modulates neural synchronization by increasing or decreasing it and induces LTP and LTD

***tES**, transcranial electrical stimulation; **tDCS**, transcranial direct current stimulation; **tACS**, transcranial alternating current stimulation; **a/c tDCS**, anodal/cathodal transcranial direct current stimulation; **LTP**, long-term potentiation; **LTD**, long-term depression; **BDNF**, brain-derived neurotrophic factor; **TrkB**, tyrosine kinase receptor B; NMDA, N-methyl-D- aspartate; **AMPA**, alpha-amino-3-hydroxy-5-methyl-4-isoxazolepropionic acid*.

Experimental evidence using a high frequency pre-synaptic stimulation protocol has showed a polarity-specificity of tDCS in the modulation of LTP induction, with anodal stimulation increasing and cathodal stimulation decreasing the amount of LTP (20). These data suggest that tDCS alone is not capable of changing synaptic strength (i.e., inducing LTP), but rather that tDCS changes the propensity of the synapse to undergo LTP. Accordingly, in the study by Fritsch and colleagues, LTP was obtained after a conditioning anodal tDCS protocol but only in the presence of concomitant synaptic activation by presynaptic inputs (21).

Neurotrophins (BDNF, NGF, NT-3, and NT-4/5) are a large family of complex proteins that regulate several functions, including neuronal survival, differentiation, synaptic function, and plasticity but also neuronal death through interaction with two types of receptors, the tyrosine kinase receptors (TrkA, TrkB, and TrkC) and the common p75NTR receptor (82). Most of neurotrophins, including BDNF, is secreted in an immature form and then converted into the mature, active form by a complex fine-regulated system of proteases (83–85). With this premise, it has been demonstrated that tDCS might increase BDNF concentration when combined with presynaptic stimulation (21) inducing LTP via BDNF/TrkB signaling (25). TrkB stimulation by BDNF also promotes long-lasting synaptic potentiation and late phase LTP requires the conversion of pro-BDNF into mature BDNF in the hippocampus (21). Moreover, enhanced LTP in animals undergoing continuous tDCS can be reduced by TrkB antagonist (86) and anodal tDCS enhances hippocampal LTP and memory via chromatin remodeling of the Bdnf gene regulatory sequence, increasing the expression of this gene (87). In addition, through TrkB/Fyn signaling, BDNF induces a phosphorylation-dependent enhancement of NMDA receptor activity that further enhances effects of tDCS on LTP (88, 89).

**Figure 1:**
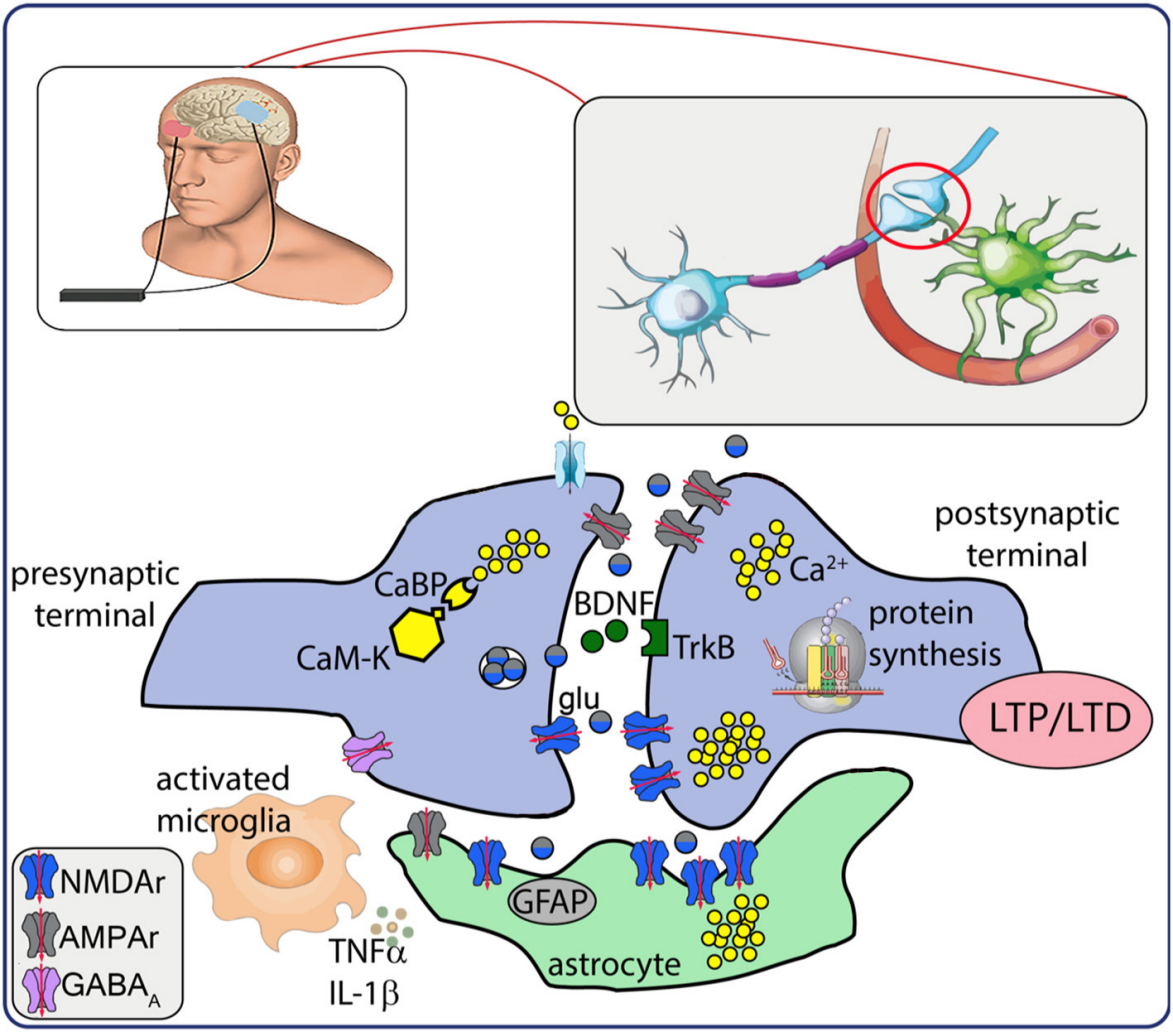
Schematic representation of neurobiological after-effects of transcranial electrical stimulation (tES). tES induces intracellular Ca^2+^ increase and activation of Ca^2+^-dependent enzymes (CaM-K). Presynaptic mechanisms result in glutamate release that activates AMPA/NMDA receptors, modulates BDNF release and interaction with TrkB receptor, responsible for a cascade of intracellular events that lead to *de novo* protein synthesis. Electrical stimulation also modulates activation of astrocytes and neuroinflammatory response. Altogether, these mechanisms may underlie the establishment of LTP/LTD. **CaBP**, Ca^2+^ binding proteins; **CaM-K**, Ca^2+^ kinases; **glu**, glutamate; **BDNF**, brain-derived neurotrophic factor; **TrkB**, tyrosine kinase receptor B; **LTP/LTD**, long term potentiation/depression; **GFAP**, glial fibrillary acidic protein; **TNFα**, tumor necrosis factor α; **IL-1β**, interleukin 1β; **NMDAr**, N-methyl-D- aspartate receptor; **AMPAr**, alpha-amino-3-hydroxy-5-methyl-4-isoxazolepropionic acid receptor; **GABA**_**A**_, gamma amino butirric acid A receptor.

The most prominent phenomena mediating LTP/LTD are the functional state of the synapse, Ca^2+^ signals and activity of NMDA glutamate receptors (74) (Figure 1). High-frequency current stimulation, in fact, induces LTP only in active synapses, which express active/open NMDA receptors, rapid expression of alpha-amino-3-hydroxy-5-methyl-4-isoxazolepropionic acid (AMPA) receptors in the postsynaptic neuron, and fast intracellular Ca^2+^ increase (90). In contrast, low-frequency, long-lasting stimulation induces small and slow rise in Ca^2+^ concentration, presynaptic internalization of AMPA receptors by phosphatase activation (that reduces glutamate sensitivity), and LTD generation (75, 76).

Studies have showed the tDCS induces changes in the direction and speed of cell migration which may be related to the shift of intracellular Ca^2+^ (77, 78) and to changes in the expression of the epidermal growth factor receptors' (EGFR) due to electrostatic effects of tDCS, ultimately contributing to long-term modulation (78).

The effects of tES on synaptic plasticity are also modulated by concomitant administration of drugs acting on neural transmissions. The dopaminergic, cholinergic, serotonergic systems all affect tDCS-induced plasticity (91) in a dose-dependent manner. For example, low dose administration of the D2/D3 agonist ropinirole abolishes plasticity (91), medium dosed ropinirole reestablishes facilitatory and inhibitory plasticity, whilst high dosage decreases facilitatory plasticity (92). Administration of low dosage or high dosage of anodal L-DOPA suppressed the plasticity induced by tDCS (79), however L-DOPA prolonged the reduction of excitability induced by cathodal tDCS (80).

Induction of plasticity through tES, however, might also arise from simultaneous stimulation of the different components of the neural circuit, from the excitatory/inhibitory synapses to different brain networks, therefore, as a result, it is important to consider the main excitatory (LTP-like) or inhibitory (LTD-like) effect of the brain stimulation. Early LTP/LTD modifications usually last for 30–60 min after induction and reflect post-transcriptional modifications of pre-existing proteins, such as protein phosphorylation, in contrast late LTP/LTD could last hours, days, and even months and require genes and proteins expression (e.g., glutamate NMDA and metabotropic receptors) (50).

In order to shed light on the pathways leading to the synthesis of new proteins, attention has been focused on the group of immediate early genes (IEGs), that are rapidly induced following neuronal activation and are thought to be involved in the maintenance of LTP (93, 94). Among IEGs, zif268 is likely to be specifically related to LTP, since it is expressed under virtually all LTP-inducing situations and shows a high correlation with the duration of LTP (95). After application of both anodal and cathodal DCS to hippocampal rat brain slices, zif268 expression was increased, pointing to a possible initial role of zif268 in a cascade of activation of other downstream target genes (20).

Abnormally high activity and hyperexcitability of some subcortical pathways, as in the case of after stroke or during central nervous system (CNS) development, may respond to tES that modulates homeostatic plasticity of the hyperexcitable tissue (96–99). The hyperexcitability is maintained because neurons receive deficient inputs and, in order to compensate, increase excitatory synaptic strength and intrinsic excitability (100, 101).

In addition, metaplastic changes are observed with the administration of tES (6). The term metaplasticity refers to a higher order form of plasticity and reflects the activity-dependent physiological changes that modulate neural plasticity (102). The history of synaptic or cellular activity influences the direction and degree of synaptic plasticity, favoring or inhibiting plasticity induction, synaptic stabilization, and homeostatic regulation of cellular activity (103). Therefore, metaplasticity acts to avoid excessive synaptic strengthening or weakening, to maintain a relatively stable equilibrium of the neural activity in space and time (homeostatic synaptic plasticity), adjusting the balance between synaptic input and neuronal firing, and to prolong the time-window for associative interactions between neural events (associative plasticity) (6). Basically, any recent neural synaptic activity will affect the ongoing activity. For example, if synaptic activity is preconditioned by applying tDCS, the application of continuous tDCS after an interval will modulate polarity which will affect performance (6). Continuous anodal tDCS has shown to facilitate performances while consecutive sessions of cathodal tDCS have shown to enhance working memory (81). Preconditioning neural networks may induce synaptic homeostatic changes that seems to be related to compensatory upregulation at post-synaptic membrane receptors due to inhibition (104, 105). This has been called as the “rebound effect” where neurons are more excitable due to initial downregulation induced by cathodal tDCS and reversed by conditioning cathodal tDCS (13).

Aberrant plasticity induced by non-invasive brain stimulation techniques has been demonstrated in many neurological and neuropsychiatric disorders including PD (106–108), dystonia (109, 110), multiple sclerosis (111), ischemic stroke (112), migraine (113), AD (114), schizophrenia (115–117), and drug addiction (103, 118).

Regarding tACS, both online and offline effects have reported to generate entertainment and neuroplasticity (45). Entertainment is where external rhythmic pattern imposes itself on the intrinsic natural pattern. Neuroplastic changes have been reported via LTP and LTD as tACS modulates neural synchronization by increasing or decreasing it (45). In summary, tES-induced mechanisms of synaptic plasticity cover different aspects of the neurobiology and neurophysiology of CNS, ranging from gene and protein expression, modulation of neurotrophins activity, and neural transmission and, finally, metaplasticity.

### Effects on Neuronal Networks and Connectivity

Polarization of the brain tissue can extend beyond the area under the electrodes (119–121) and it may have a functional effect also on distant interconnected neural networks (122, 123). Anodal tDCS of the premotor cortex, for example, increases the excitability of the ipsilateral motor cortex (124) and stimulation of the primary motor cortex has inhibitory effects on contralateral motor areas (125). EEG studies support these findings, showing that stimulation of frontal areas induces all-brain synchronous changes of the oscillatory activity (126, 127). Altered prefrontal oscillations and brain synchronization have been reported by magnetoencephalography (MEG) and EEG study in AD, showing functional disconnection between prefrontal cortex and hippocampus and changes of network connectivity (128–130).

Functional connectivity of cortical networks increased within motor, premotor, and somatosensory areas after anodal tDCS, inducing significant intra and interhemispheric connectivity changes, as revealed by analysis of EEG frequency bands (131).

Brain areas interact mutually creating a complex network that underlie higher brain functions and neural synchronization represents an essential system to coordinate cortico-cortical and cortico-subcortical areas (132, 133). A combined tDCS-fMRI study revealed that after active stimulation functional connectivity showed an increased synchrony in the anti-correlated network (that includes DLPFC) and reduced in the default mode network (DMN) components, thus suggesting a functional reconfiguration of intrinsic brain networks after tDCS (134). This could represent a putative mechanism for tDCS-induced improvement of cognitive functions (134). In addition, using fMRI, anodal tDCS was also shown to modulate functional connectivity of cortical (70), cortico-striatal and thalamo-cortical motor pathway (135). To better grasp the precision of tES, stochastic resonance should be underlined. The concept of stochastic resonance attempts to highlight the importance of wide range of affects due to TES. The electric field can be considered as noise and when added to non-linear systems may enhance or disrupt the state of signal and the noise introduced (136–138). Since the after-effects are not focal but global, the dynamic interactions will modulate not only particular group of neurons but also induce global effects thus affecting neurons near their discharge threshold, thus facilitating or inhibiting a subthreshold signal which will produce two different polarized after effects (138).

See Table 4 for a summary of the main tES studies and results on neuronal networks and connectivity.

**Table 4:** tES after-effects on neuronal networks and connectivity.

**References/Study**	**Methodology tES**	**Targets**	**Main results**
Boros et al. (124); Vines et al. (125)	tDCS - aDCS	Motor cortex	aDCS of premotor cortex increases the excitability in ipsilateral motor cortex. Stimulation of primary motor cortex has inhibitory effect on contralateral motor area
Polanía et al. (131)	tDCS	Motor/premotor/somatosensory areas	Functional connectivity of cortical networks increased with aDCS with intra/interhemispheric connectivity changes
Peña-Gómez et al. (134)	tDCS	Default mode network and DLPFC	tDCS increased synchrony in anti-correlated network and reduced in default mode network
Stagg et al. (55)	tDCS	Cortical/cortico-striatal/thalamo-cortical motor pathways	tDCS modulates functional connectivity of cortical, cortico-striatal and thalamo-cortical motor pathways
Fertonani and Miniussi (138)	tACS/tDCS	–	tES induces stochastic resonance which affects neuronal groups and induces wide range of global effects by facilitating or inhibiting a subthreshold signal

***tES**, transcranial electrical stimulation; **tDCS**, transcranial direct current stimulation; **tACS**, transcranial alternating current stimulation; **a/c tDCS**, anodal/cathodal transcranial direct current stimulation; **DLPFC**, dorsolateral prefrontal cortex*.

### Effects on Glial Cells and Neuroinflammation

The relevance of glial biology cannot be neglected to understand the complexity of the CNS and the comprehensive mechanisms and effects of tES. The significance is clinically appealing as glial cells create a wide neuro-glial network for rapid inter-cellular long-range signaling (73) and are early affected in many CNS disorders. Although the glial cells have attracted limited interest for decades, it is only recently that studies have focused on their role in maintaining synaptic homeostasis and modulating synaptic plasticity in health and disease (139). Astrocytes and microglial cells are in close proximity with synapses as they directly modulate synapse formation and elimination (140). The loss of integrity of these supportive cells is the trigger of neurodegenerative disorders (141–143). Initially it was believed that AD was consequentially due to Aβ oligomers and fibrils that accumulate and inflammation. However, now it has been demonstrated that glial cells drive the synaptic loss in AD (144–147). In addition, glial mediated synapse formation may impair synaptic turnover and homeostasis which disrupts synaptic plasticity. Reactive gliosis is a process of hypertrophy and proliferation of glial cells in response to an insult such infection/trauma/neurodegenerative disorders (140, 148). This is proceeded by release of chemokines, cytokines and neurotrophic factors that have both neuroprotective (M2-like microglia) and neuroinflammatory effect (M1-like microglia) (84). This leads to a simultaneous process of neural damage and synaptic loss with tissue remodeling and phagocytosis.

To our best knowledge, there are no reports regarding the activity of tACS on glial cells. Significant after-effects of tDCS on glial cells function and plasticity are reported by several groups in the last years (see Table 5). This is supported by the fact that astrocytes possess voltage-gated channels and transporters that are sensitive to changes of membrane potential (152, 153). Administration of tDCS has shown to cause a surge in Ca^2+^ in cortical astrocytes that is correlated to an overexpression of the glutamate NMDA receptor (154). Evidence suggests that tES modulates the activity of microglia cells but also the neuroinflammatory response, triggering both pro-inflammatory and anti-inflammatory reaction (149). Cathodal and anodal tDCS produce microglial activation as indicated by the increase of Iba-1, an immunostaining marker of activated microglia (150). High voltage anodal and cathodal tDCS was demonstrated to trigger an inflammatory response in the microglial cell line BV2, showing increase of cyclooxygenase 2 (COX-2) expression, leukocyte transmigration through blood brain barrier (32, 149). On the other hand, there was decrease of tumor necrosis factor-alpha (TNF-α) in rat hippocampus after anodal tDCS of parietal cortex (151). Modulation of the neuroinflammatory reaction is relevant because microglia activation can be beneficial as well as detrimental for neural tissue depending on the time of activation. This is clinically relevant in the case of ischemic stroke, because tDCS can activate innate immune response and attract neural stem cells. *In vitro* experiments suggest that cathodal tDCS, delivered for 5 days, can induce cell proliferation and attract neural crest stem cells (149), forming a reservoir of neurotrophic factors which improved functional recovery. In addition, tDCS has also been shown to influence astrocytes by aligning them perpendicular to the electrical field in both *vitro* and *in vivo* (155–157).

**Table 5:** tES after-effects on glial cells and inflammation.

**References/Study**	**Methodology tES**	**Targets**	**Main results**
Rueger et al. (149)	DCS	Microglial cells	tES produces both proinflammatory and anti-inflammatory reactions
Pikhovych et al. (150)	tDCS - cDCS - aDCS	Microglial cells and Iba-1	cDCS and aDCS cause microglial activation with increase in Iba-1 markers
Rueger et al. (149); Pelletier and Cicchetti (32)	High voltage DCS - cDCS - aDCS	Microglial cell BV2	High voltage aDCS and cDCS induces activation of microglial cells BV2 with increased expression of COX-2 (cyclooxygenase 2) and leukocyte transmigration
Spezia Adachi et al. (151)	DCS - aDCS	Hippocampal neurons	aDCS of parietal cortex decreased tumor necrosis factor alfa (TNF-α) in the rat hippocampus
Rueger et al. (149)	DCS	Neural crest stem cells	5-day cDCS induced cell proliferation and attracted neural stem cells

***tES**, transcranial electrical stimulation; **DCS**, direct current stimulation; **a/c DCS**, anodal/cathodal direct current stimulation*.

Due to the remarkable connectivity of astrocytes and their pivotal role in neuronal connectivity, non-invasive brain modulation may have profound neurobiological effects (158).

## Potential Clinical Applications of Current Stimulation

Efficacy of tES in the clinical setting has been supported by many experimental works and clinical reports that has demonstrated a long-lasting efficacy in many neurological and psychiatric conditions (5). Despite neurobiological mechanisms have not been yet fully understood, it is supposed that tES-induced modulation of cortical excitability through changes in cell firing rate could pave the way for future therapeutic applications (159).

Application of tACS in the clinical setting is very limited and largely implemented in the psychiatric settings (160, 161). Accordingly, tACS was shown to successfully manipulate auditory hallucinations in schizophrenia by decoupling interhemispheric connectivity and, when administered to schizophrenic patients to the left dorsolateral prefrontal cortex and posterior parietal region in theta frequency (6 Hz), improved working memory tasks (162). Moreover, 40 Hz tACS induced improvement/remission of symptoms in major depression (163) and obsessive compulsive disorder (164) by modulation of EEG-gamma frequency bands. Enhancement of gamma band power connectivity by tACS was also effective in patients with AD and mild cognitive impairment (165, 166).

Experimental and clinical research with tDCS has been widely explored for its ability to suppress neuronal hyperexcitability or by enhancing inhibition (167). While cathodal tDCS reduces cortical excitability due to neuronal hyperpolarization, anodal tDCS causes an increase in cortical excitability and promotes neuronal depolarization (168). These neurobiological effects might be the substrate to counteract the temporoparietal hypoactivity (atrophy, reduced metabolic rate, and perfusion) reported in AD, suggesting an innovative therapeutic strategy (169).

In an experimental rat model of stroke, tDCS induced a dramatic increase in spine density of cortical neurons at the site of infarct, indicating that it may promote neural plasticity after stroke (170). Accordingly, tDCS was found to down-regulate the elevated hemichannel pannexin-1 mRNA expression after brain ischemia (thus reducing membrane permeability), but also increase the expression of MAP-2 and GAP-43 proteins, allowing axons to regrow at the infarcted site through the glial scar and redevelop their functions (171). Interestingly, tDCS performed within 3 days after stroke did not improve motor function, in contrast when performed 7–14 days after stroke resulted in more pronounced motor function improvement, thus identifying an optimal time-window for tDCS therapy after stroke (171).

In patients with multiple sclerosis (MS) that received tDCS, MRI detected (1) increased cerebral metabolic rate of oxygen (CMRO_2_), an indicator of the overall brain/neural activity, and (2) a reduced neuronal reactivity (172).

Seizures are described as a result of an increased excitability and inefficient inhibitory control in foci with altered neuronal homeostasis (72, 173, 174). In the recent years, many works have reported the efficacy of tES in the treatment of drug-resistant seizures. Authors observed an enhanced neuronal plasticity and synaptic reorganization after tES (100). For example, it has been reported that temporal lobe epilepsy responded to tES of hippocampus (101) and low frequency tACS applied over the epileptic foci might reduce interictal and ictal activities in epileptics (175). Moreover, experimental evidence in a rat model of focal epilepsy demonstrated that cathodal tDCS has an anticonvulsant effect through increase of the localized seizure threshold that outlasted the stimulation (176). Similar results were confirmed on a refractory pediatric epileptic patient with focal cortical dysplasia who was treated with cathodal tDCS and experienced marked reduction in the frequency of seizures (177). Along with this, cathodal tDCS was reported to prevent the loss of GABAergic inhibition, which provokes seizures after pentylenetetrazol administration, thus proposing a new antiepileptic mechanism (178). These results, therefore, have posed the basis to the clinical combination of the cathodal tDCS with GABA-agonist antiepileptic drugs (AEDs), such as benzodiazepines, valproic acid, felbamate, topiramate, and barbiturates, in order to increase the antiepileptic stimulation effect.

Application of tDCS is not limited to the cerebral cortex and its disorders but also for the modulation of the excitability in the cerebellum and spinal cord. Since pharmacological approaches to treat cerebellar diseases are still lacking, tES might represent a new potential therapeutic approach that is yet to be explored. The mechanisms behind the neurophysiological effects of tDCS applied over cerebellum have not been extensively researched as compared to cerebral cortex. However, it could be inferred that ionic gradient shifts, cellular activation and inhibition, modulation of neurotransmission may occur in the same way (179). Evidence suggests that cerebellar cathodal tDCS decreases the inhibitory tone of cerebellum on primary motor cortex while anodal tDCS increases it, likely through a specific modulation of dentate-thalamo-cortical connections (21). TDCS also modulates cerebellum-dependent motor learning: anodal tDCS improved the performance in a locomotor adaptation task (180). Mechanisms need to be further explored, however it has been hypothesized that anodal tDCS may broaden the availability of Purkinje cells for learning or increase the dynamic range of these cells, whereas cathodal tDCS may reduce the excitability of Purkinje cells (181). The effects of tDCS on cerebello-motor connectivity were studied in 20 patients with ataxia with administration of cerebello-spinal tDCS (179). Improvement in ataxia was reported and was associated with restoration of motor cortex excitability and cerebellar-brain inhibition.

Application of spinal tDCS is very limited but the preliminary results are extremely interesting. It has been reported that spinal anodal tDCS reduces the amplitude of laser evoked potentials of stimulated Aδ fibers (182) and increases cortico-spinal excitability in a polarity-independent manner (183). While spinal anodal tDCS inhibits the ascending pathways and enhances the reflex circuitry, the spinal cathodal tDCS enhances the activity of ascending pathways and suppresses the reflex circuitry in humans (181). Since there is involvement of the ascending and descending pathways, the glutamatergic, GABAergic and glycinergic systems should be involved in modulating the spinal plasticity (181). The effects of this kind of stimulation can vary in response to several factors including intensity, polarity and direction (184) but also through modulation of the voltage-gated Ca^2+^channels in the spinal motor neuron dendrites (185). Altogether, these preliminary results demonstrate the ability to modulate spinal plasticity with electrical current stimulation, paving the way for new therapeutic strategies in neurological disorders with impaired spinal excitability.

## Challenges and Future Directions

To date, despite the undisputed role of tES in experimental settings in humans as a tool to “switch on/off” specific brain regions that are supposed to be involved in several higher brain functions, its translation into clinical settings is still far to be reached due to the difficulty in producing clinically significant effects in the majority of subjects/patients. This is largely due to the lack of a full comprehension of both the neurobiological bases of tES and the specific neuropathological mechanisms of disease. There are still few data on the possible clinical efficacy of prolonged/repeated protocols of stimulation that might produce persistent changes in synaptic efficacy that cannot be achieved by a short-lasting intervention. In this context, successful trials of prolonged tES protocols could eventually be translated into invasive implants of cortical electrodes for chronic stimulation. Finally, tDCS shows lack of selectivity that might influence different cortical circuits and produce side effects that counteract the effects responsible for the therapeutic action. Therefore, optimizing protocols, electrode size and intensity of stimulation should help to overcome these technical limitations that impedes a tailored approach to the patient and disease.

## Author Contributions

SK: acquisition, analysis, and interpretation of data for the work and drafting the manuscript. FR: supervising and editing the manuscript and final approval of the manuscript to be submitted. VD: conception and design of the work, supervising and editing the manuscript, and final approval of the manuscript to be submitted. MP: critical supervision, manuscript editing, and final approval of the draft to be submitted. GC: conception and design of the work, analysis and interpretation of data, revising the manuscript, and final approval of the manuscript to be submitted. All authors contributed to the article and approved the submitted version.

## Conflict of Interest

The authors declare that the research was conducted in the absence of any commercial or financial relationships that could be construed as a potential conflict of interest.

## Abbreviations

*tES*: transcranial electrical stimulation.
